# From Atlas to Algorithms: Bridging Humanism and Education With AI

**DOI:** 10.1007/s11606-026-10343-4

**Published:** 2026-03-12

**Authors:** Adi Friedman

**Affiliations:** Indiana University School of Medicine, Indianapolis, IN USA

The C7 vertebra, the lowest in the cervical spine which protrudes just under the skin at the base of the neck, was for centuries referred to as “Atlas,” a reference to the titan who was condemned by Zeus to bear the world upon his shoulders for all eternity after leading an unsuccessful rebellion against the Olympian gods. All this changed in 1522, when the famous anatomist Jacopo Berengario in his textbook *Isagogae Breves* renamed the C1 vertebra as Atlas.^1^ What prompted this shift? Berengario intended to draw an elegant (and more anatomically accurate) parallel between the world foisted on Atlas’ shoulders and the human head supported by the C1 vertebra. This choice also reflected the spirit of Renaissance humanism that defined his era. In that period, marked by a revival of classic learning and faith in human reason, Berengario likely imagined Atlas not as a figure condemned to bear the burden of an indifferent world upon his shoulders, but as a symbol of mankind’s ability to understand and reshape that world through the power of reason.

Today we are witnessing a paradigm shift of similar magnitude with the technological revolution in medicine. In what feels like a sci-fi plot, technology and increasingly AI now challenge the primacy of the human intellect not only in scientific discovery, but in how we think, create, and relate to one another. This abrupt change threatens to disrupt traditional medical learning by offering an alluring alternative to the dedication once required to master diagnostic and clinical skills. Over time, it risks pushing human enterprise to the periphery and weakening the foundational physician-patient relationship.

For this reason, entering medical school today is both disorienting and exciting. As my classmates and I experience the rapid transformation of medicine in real time, we often feel disconnected from the centuries-long tradition of humanism in Western medicine that is rooted in profoundly human interactions between healers and their patients. We are left to grapple with the tension inherent in mastering an ancient profession steeped in knowledge of the human body while the profession itself evolves at light speed.

The best way to resolve this tension may lie in reinforcing the marriage between medical education and humanism. My introduction to anatomy provides an excellent example. The essential structures of the human body have remained the same since time immemorial and memorizing them remains a tedious rite of passage for every medical student. I experienced this in my first month of school after spending many frustrating hours trying to learn each bone and every muscle, with their origins and insertions. What I quickly realized I lacked was a humanistic framework to help assimilate this massive amount of information in a more meaningful way.

I could have gone to the library to address this issue, but instead I turned to ChatGPT and asked: “what is the etymological origin of the Atlas vertebra?” Within moments I was exploring centuries of rich medical history with simple prompts. I found that my peers from varying academic backgrounds embraced this strategy with equal enthusiasm. While studying together, an interesting anatomical name would spark lively conversation and leave us with a refreshed curiosity. We began to see human anatomy through the eyes of the great anatomists preceding us—individuals who not only systematized the body’s immense complexity but also captured its magnificence through rich metaphors and knowledge of the world they inhabited.

This shift in perspective transformed what began as an exercise in rote memorization into a fascinating exploration of a world brought to life by AI. I learned of the many anatomical parts such as the coracoid process (“raven-like”) of the scapula, the lumbrical (“earthworm”) muscles of the hands and feet, and the cauda equina (“horse’s tail”) of the spinal cord that drew inspiration from the animal kingdom. Organic forms were also integrated into anatomical nomenclature with names such as the foliate papilla (“leaf-shaped”), the lunate (“moon-shaped”), and pisiform (“pea-shaped”) bones of the wrist, and the pineal (“pinecone”) gland. Anatomists incorporated cultural references as well, as seen with the xyphoid (“sword-shaped”) process, the tibia (“flute”), the mesosalpinx (“mesentery of the trumpet”) of the uterine tube, and the sella turcica (“Turkish saddle”) of the sphenoid bone. They even injected humour into their work, with for example the storage and maturation site for spermatozoa located on the back of each testicle known as the epididymis (“upon the twins”).

When prompted, AI also provided a historical and cultural depth that resonated with me. For example, I learned that the name “hamstring” was derived from the butcher’s practice of hanging animal carcasses by the tendons of these muscles; the sartorius (“tailor”) muscle from the posture tailors adopted while sewing (with their knees flexed and their legs laterally rotated); the groove on our upper lips known as the philtrum (“love-charm”) from its sensual appeal; the vagus (“wandering”) nerve from its long and winding path from the brainstem to the abdomen; and the pudendal (“to be ashamed”) nerve, which innervates the genitalia and anus, from enduring cultural attitudes towards our most private parts.

In this era of rapid technological advancement, students would greatly benefit from more integration of the profession’s humanistic foundations into medical education. Just as Berengario’s renaming of Atlas reflected a shifting understanding of man’s place in the world, the inevitable technological transformation of medicine invites us to consider how we can preserve—or even enhance—the humanistic aspects of medicine while embracing change. When used thoughtfully, generative AI can serve as a powerful tool for doing so by reconnecting medical trainees and physicians to the inspiring roots of medicine, fostering a deeper sense of awe for the human body, and even translating that passion into more holistic, patient-centered care. In doing so we can ensure that upholding the humanistic foundations of medicine remains a human responsibility—supported but never borne by AI.

## References

[1] **Jackowe D, Biener M.** Atlas and Talus. J Anat. 2022;240(6):1174–1178. 10.1111/joa.13613. 10.1111/joa.13613 PMC9119609 34914100

